# Adolescent pregnancy as a blind spot in RSV prevention: rethinking maternal and infant immunization policies

**DOI:** 10.1093/eurpub/ckag095

**Published:** 2026-06-12

**Authors:** Manuela Chiavarini, Paolo Bonanni, Angela Bechini, Sara Boccalini

**Affiliations:** Department of Health Sciences, University of Florence, Florence, V.le Morgagni, Florence 50121, Italy; Department of Health Sciences, University of Florence, Florence, V.le Morgagni, Florence 50121, Italy; Department of Health Sciences, University of Florence, Florence, V.le Morgagni, Florence 50121, Italy; Department of Health Sciences, University of Florence, Florence, V.le Morgagni, Florence 50121, Italy

We have read with great interest the article by Lubrano *et al.* [1] entitled “Italian survey on maternal acceptance and views on RSV vaccination during pregnancy,” which explores awareness, knowledge, and acceptance of vaccines recommended during pregnancy, including pertussis, influenza, and respiratory syncytial virus (RSV). Their findings should be interpreted within the broader policy framework in which maternal immunization—endorsed by the World Health Organization—is positioned as one of the key strategies to reduce severe respiratory infections in early infancy [2]. Despite this high reported willingness to vaccinate, immunization coverage during pregnancy remains suboptimal in countries such as Italy, reflecting structural, organizational, and communication constraints rather than lack of acceptance. This persistent intention–uptake gap points to the need for a paradigm shift from demand-oriented approaches to delivery-focused models, in which vaccination is systematically embedded within antenatal care pathways and implemented as a standard component of maternal services [3]. At the same time, the growing availability of other infant-targeted passive immunization strategies—particularly long-acting monoclonal antibodies such as nirsevimab—introduces new dimensions for maternal and child health policies. Although maternal vaccination is often preferred in survey settings, real-world programs have demonstrated high uptake of monoclonal antibody administration in early infancy, including in Italy. This divergence suggests that preventive behaviors are not solely preference-driven but are strongly influenced by how interventions are operationalized, financed, and integrated into healthcare systems. From a public health perspective, this underscores the need to move beyond parallel or competing strategies and toward coordinated, life-course immunization frameworks that align maternal and infant interventions, reduce fragmentation in service delivery, and ensure equitable access to prevention across different population groups [4, 5].

Against this background, the case of adolescent pregnancy illustrates how these unresolved tensions between maternal and infant prevention strategies become most visible when equity, regulatory eligibility, and access to care intersect. Pregnant adolescents represent a structurally under-recognized group within maternal immunization policies. In addition, early motherhood is frequently associated with social vulnerability, lower health literacy, and reduced access to preventive services. This context creates a situation of double vulnerability, where biological and social risks intersect with structural barriers to care [6].

Although their absolute numbers remain limited in Europe [7], their risk profile warrants specific consideration within equity-oriented public health frameworks. In this context, the current regulatory landscape for RSV prevention reveals a critical gap: the only vaccine authorized in Europe for maternal immunization, Abrysvo, is approved exclusively for individuals aged 18 years and older, thereby excluding pregnant adolescents from access to antenatal vaccination strategies (Abrysvo European Medicines Agency (EMA); https://www.ema.europa.eu/en/medicines/human/EPAR/abrysvo (17 March 2026, date last accessed)). While this threshold originates from regulatory authorization based on available trial data, it results in an explicit exclusion of adolescents from a key preventive strategy. This regulatory threshold effectively creates a “blind spot” in prevention policies, where a subgroup potentially at higher risk of adverse maternal and neonatal outcomes is not covered by the primary immunization tool, in that way generating a clear form of age-based inequity in access to prevention [2]. To contextualize this regulatory gap within the broader maternal immunization landscape, the main vaccines currently recommended during pregnancy and their regulatory characteristics are summarized in Table 1.

**Table 1.** Main vaccines recommended during pregnancy: regulatory characteristics and relevance to adolescent pregnancy.

Vaccine	Type of vaccine	Authorized age	Use during pregnancy	Potential issue in adolescents
Tdap (tetanus–diphtheria–pertussis)	Inactivated vaccine	Different vaccine formulations for various age groups	At each pregnancy, in the second or third trimester of pregnancy, according to national official recommendations	No age-related barrier
Influenza	Inactivated influenza vaccine (IIV)	≥6 months	In any trimester of pregnancy	No age-related barrier
COVID-19	mRNA vaccines	≥6 months	In the second or third trimester of pregnancy, according to national official recommendations	No age-related barrier
RSV	Recombinant prefusion F protein vaccine	≥18 years	Single dose at 24–36 weeks of gestation, according to national official recommendations	Pregnant adolescents fall outside current age-based authorization for maternal RSV vaccination, leaving a gap in RSV protection for both mothers and their infants.

As a consequence, infants born to adolescent mothers can only be protected through alternative passive immunization strategies, notably the administration of long-acting monoclonal antibodies such as nirsevimab. While effective, this infant-targeted approach shifts the preventive focus from antenatal to postnatal care and relies on timely access to pediatric services, which may itself be unevenly distributed. Importantly, this differentiation in preventive pathways is not driven by distinct clinical indications or evidence of differential effectiveness, but rather by regulatory constraints linked to age-specific authorization. This dynamic raises important questions regarding program design and equity: rather than offering interchangeable options, current systems may inadvertently stratify protection pathways based on maternal age, reinforcing disparities in access to prevention. The limited integration between maternal and infant immunization strategies further underscores how organizational factors can shape health outcomes independently of clinical efficacy [8–10].

More broadly, the marginal position of pregnant adolescents reflects a systemic gap at the intersection of regulatory science, clinical research, and public health policy. Maternal immunization programs have largely developed around adult populations, with adolescents frequently excluded from clinical trials and, consequently, from evidence-based recommendations. This lack of evidence does not reflect an absence of need but rather an evidence-generation gap that translates into regulatory ineligibility and programmatic exclusion. This dynamic creates a cycle of “evidence invisibility” that reinforces continued neglect in both regulatory and programmatic domains. As a consequence, a misalignment emerges between available evidence, regulatory frameworks, and the actual health needs of a vulnerable population such as pregnant adolescents. Addressing this gap requires a dual approach: generating age-inclusive evidence on vaccine safety and effectiveness during adolescent pregnancy and redesigning immunization policies to explicitly incorporate vulnerable subgroups within maternal and child health strategies. In line with the priorities of the World Health Organization, future research and policy development should aim to ensure that maternal immunization frameworks are not only effective at the population level but also equitable, inclusive, and responsive to the needs of those at the margins of current prevention systems [2, 6].

In conclusion, the case of pregnant adolescents exposes a structural blind spot in maternal immunization policies: a mismatch between regulatory frameworks and the populations they are intended to protect. In the context of RSV prevention, the age restriction applied to Abrysvo effectively excludes adolescents from antenatal vaccination, leaving them reliant on postnatal alternatives such as nirsevimab and creating age-differentiated pathways of protection in a group already facing heightened vulnerability. This raises a fundamental policy question: are current strategies truly evidence-based or simply evidence-limited? When the absence of data results in regulatory exclusion, it risks becoming a proxy for inequity. The systematic exclusion of pregnant adolescents—despite their vulnerability—calls into question the fairness and public health rationale of existing frameworks. If maternal immunization is to fulfill its preventive and equity goals, adolescents can no longer remain outside both the evidence base and the policy lens.

## Data Availability

Not applicable, as no datasets were generated or analyzed during the current study.
